# Headless Compression Screw Fixation Alone for Lunate Coronal Fractures in Kienböck Disease

**DOI:** 10.1111/os.70410

**Published:** 2026-08-20

**Authors:** Jiangchao Zhang, Binzhi Zhao, Deshui Yu, Liping Xu, Ge Xiong

**Affiliations:** ^1^ Department of Hand Surgery Peking University Fourth School of Clinical Medicine Beijing China; ^2^ Department of Hand Surgery Beijing Jishuitan Hospital, Capital Medical University Beijing China; ^3^ Department of Radiology Beijing Tongren Hospital, Capital Medical University Beijing China

**Keywords:** coronal fracture, fixation, Kienböck disease, treatment, union

## Abstract

**Objective:**

Lunate coronal fractures in Kienböck disease remain challenging to manage, and various reconstructive approaches have been described. The purpose of this study was to evaluate the outcomes of headless compression screw fixation alone in management of the lunate coronal fractures in Kienböck disease.

**Methods:**

Twelve patients (seven females and five males; mean age 36.9 years) with lunate coronal fractures who underwent headless compression screw fixation between 2017 and 2025 were included in this retrospective study. Clinical and radiographic assessments were performed preoperatively and at final follow‐up. The clinical evaluations included visual analog scale (VAS), Quick Disabilities of the Arm, Shoulder and Hand (QuickDASH) score, Modified Mayo Wrist (MMW) score, active range of motion, and grip strength. Radiographic parameters included the radioscaphoid angle, Ståhl index, and Nattrass index. Comparisons between preoperative and final follow‐up continuous variables were performed using the paired *t*‐tests or Wilcoxon signed‐rank tests, as appropriate.

**Results:**

The median clinical follow‐up was 56.8 months (range: 12–101 months). Radiographic follow‐up was available in 11 patients, and the median imaging follow‐up was 50.9 months (range: 12–99 months). One patient declined further imaging examinations due to complete symptom resolution. Among patients with available imaging, all coronal fractures achieved union. The average time to bone union was 7.6 ± 1.2 months. Significant improvements in wrist pain, QuickDASH score, MMW score, range of motion, and grip strength were observed after surgery. However, changes in radioscaphoid angle, Ståhl index, and Nattrass index showed no significant difference at final follow‐up.

**Conclusion:**

Headless compression screw fixation alone appears to be a reasonable treatment option for lunate coronal fractures in Kienböck disease in the absence of radiocarpal or midcarpal arthritis and without substantial lunate comminution.

## Graphical abstract

The optimal management of Kienböck disease with lunate coronal fractures remains controversial. Previous studies advocated excision of the fractured lunate followed by scaphotrapeziotrapezoidal (STT) fusion or proximal row carpectomy. In this study, headless compression screw alone achieved fracture healing and satisfactory clinical outcomes in selected patients.

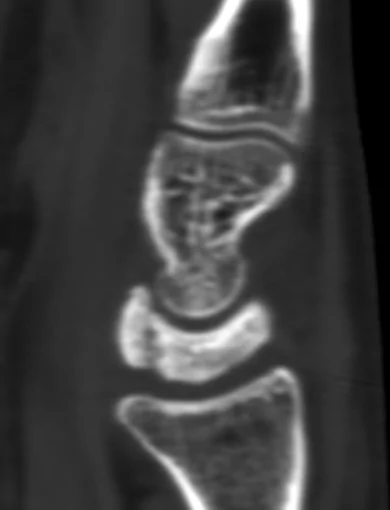

## 1 Introduction

Kienböck disease is an uncommon, progressive disorder characterized by osteonecrosis of the lunate that may progress to lunate fracture and subsequent collapse, ultimately leading to carpal instability and advanced wrist arthritis [1, 2]. Although this condition has been recognized for more than a century, its exact etiology and pathogenesis remain poorly understood, and there is still no consensus on the optimal treatment approach. Consequently, it continues to pose a significant challenge in hand surgery [3, 4].

Lunate fractures in Kienböck disease are well recognized, and several fracture patterns have been described. Among these, the most common reported pattern is the coronal fracture [5]. Previous studies have suggested that this type of fracture tends to be associated with poor clinical outcomes and often fails to achieve union. Accordingly, palliative procedures such as limited fusion and proximal row carpectomy were commonly recommended in the past [6, 7, 8]. More recently, however, lunate coronal fractures have been reported to be salvaged with reconstructive procedures. These reconstructive strategies typically involve a combination of procedures, including radial shortening osteotomy or other unloading osteotomies, vascularized or nonvascularized bone grafting, with or without internal fixation, and the reported outcomes have remained inconsistent [9, 10, 11, 12, 13, 14, 15].

As a result, an important clinical question remains whether stable fixation alone may be sufficient for lunate coronal fractures in Kienböck disease without the need for additional procedures. To our knowledge, the outcomes of headless compression screw fixation alone for these fractures have not been reported. Therefore, the aims of the current study were: (i) to evaluate the postoperative clinical outcomes after headless compression screw fixation alone; (ii) to assess fracture union and changes in radiographic parameters after surgery; and (iii) to explore whether this direct fixation strategy may represent a feasible treatment option for lunate coronal fractures in Kienböck disease. We hypothesized that this approach would achieve fracture healing, relieve pain, and maintain or modestly improve wrist function.

## 2 Methods

### 2.1 Patients Selection

This study was approved by our institutional ethics committee (K2025186) and written informed consent was obtained from each patient. Between 2017 and 2025, 12 patients of Kienböck disease with lunate coronal fractures who underwent headless compression screw fixation were retrospectively reviewed. The diagnosis was established based on medical history, physical examinations, and imaging findings. The indication for headless compression screw fixation alone was the absence of radiocarpal or midcarpal arthritis and substantial lunate comminution on preoperative imaging.

Patients with a history of wrist trauma, systemic bone disorders, prior wrist surgery, or who were lost to follow‐up were excluded. The cohort included seven women and five men, with a mean age of 36.9 years (range, 16–60 years). All patients had unilateral involvement, with nine right and three left wrists. All cases were right‐hand dominant. Preoperative Lichtman classification was stage II in one patient and stage IIIC in 11 patients.

### 2.2 Surgical Technique and Postoperative Follow‐Up

All fractures were treated with headless compression screw fixation, and all procedures were performed in the operating room by a senior hand surgeon. The surgical approach was selected based on the fracture configuration. A volar approach was used in nine patients and a dorsal approach in three. After exposure of the lunate, fibrous tissue or sclerotic bone at the fracture interface was debrided as needed to facilitate fracture reduction and create a more favorable environment for healing. A guidewire was then inserted across the fracture under fluoroscopic guidance. After confirmation of satisfactory guidewire position, the fracture was reduced, and a 2.5‐mm Acutrak headless compression screw (Acumed LLC, Hillsboro, OR, USA) was inserted across the fracture. Screw trajectory and final position were confirmed with intraoperative fluoroscopy. The screw was retained after fracture healing and was not routinely removed unless implant‐related complications occurred. No concomitant unloading osteotomy or bone grafting was performed. Postoperatively, the wrist was immobilized in a short‐arm cast for 8 weeks, followed by initiation of active range of motion exercise. Patients were instructed to avoid heavy lifting until satisfactory lunate fracture healing was confirmed radiographically. They were followed at regular intervals in outpatient clinic visits, with telephone contact used as needed. Clinical and radiographic assessments were performed preoperatively and at final follow‐up. Clinically, wrist pain at rest was assessed using the visual analog scale (VAS), and the wrist function was evaluated using the Quick Disabilities of the Arm, Shoulder and Hand (QuickDASH) score and a Modified Mayo Wrist (MMW) score. Active range of flexion‐extension motion was measured with a goniometer and grip strength was measured and expressed as percentages of the contralateral hand. Radiographic assessments included measurement of the radioscaphoid angle, Ståhl index, and Nattrass index [16].

### 2.3 Statistical Analysis

Normality of continuous variables was assessed using the Shapiro–Wilk test. Data with a normal distribution was expressed as mean and standard deviation (SD), and for those that did not, the median (interquartile range) was used. Categorical variables were represented as frequency. Comparisons of continuous data before surgery and at final follow‐up were performed using the paired *t* test for normally distributed data and Wilcoxon signed‐rank test for nonnormally distributed data. Categorical variables were compared using Fisher exact test. *p* < 0.05 was considered statistically significant.

## 3 Results

### 3.1 Follow‐Up and Fracture Union

The median clinical follow‐up was 56.8 months (range: 12–101 months). Radiographic follow‐up was available in 11 patients, with a median imaging follow‐up of 50.9 months (range: 12–99 months). The average time to bone union in 11 patients was 7.6 ± 1.2 months. One patient declined further imaging examinations because her symptom had completely resolved, and she returned to her previous work. Therefore, she was assessed by telephone interview only. Comparisons of clinical and radiographic parameters before surgery and at final follow‐up were shown in Table 1.

**TABLE 1:** Comparison of clinical and radiographic parameters preoperatively and at final follow‐up.

Parameter	Before surgery	At final follow‐up	*p*
Pain in VAS	7.5 ± 2.1	1.3 ± 1.1	< 0.05
Grip strength	41.3 ± 10.9	76.3 ± 15.5	< 0.05
ROM (°)	43 ± 18	94 ± 24	< 0.001
QuickDASH score	44.1 ± 9.2	6.2 ± 3.1	< 0.001
MMW score	33.3 ± 9.4	78.4 ± 9.7	< 0.001
Radioscaphoid angle (°)	45 ± 13	47 ± 12	0.693
Ståhl index	0.51 ± 0.13	0.50 ± 0.17	0.926
Nattrass index	1.52 ± 0.42	1.50 ± 0.59	0.766

### 3.2 Clinical Outcomes

Wrist pain improved significantly after surgery, with the mean VAS score at rest decreasing from 7.5 preoperatively to 1.3 at final follow‐up (*p* < 0.05). The mean total wrist active flexion‐extension arc was 43° ± 18° before surgery and 94° ± 24° after surgery (*p* < 0.001). The grip strength improved significantly from 41.3% of the contralateral hand preoperatively to 76.3% at final follow‐up (*p* < 0.05). Before surgery, the mean QuickDASH score and MMW score were 44.1 and 33.3, respectively, and improved to 6.2 and 78.4 (*p* < 0.001).

### 3.3 Radiographic Outcomes

Among the 11 patients with available mid‐ to long‐term follow‐up imaging (≥ 12 months), all coronal fractures achieved union at final follow‐up (Figures 1, 2, 3). There were no intraoperative nor postoperative complications. No progression in Lichtman stage was observed on follow‐up imaging. The mean radioscaphoid angle was 45° ± 13° preoperatively and 47° ± 12° at final follow‐up (*p* = 0.693). The Ståhl index remained unchanged (0.51 ± 0.13 vs. 0.50 ± 0.17; *p* = 0.926), and the Nattrass index was similar preoperatively and at final follow‐up (1.52 ± 0.42 vs. 1.50 ± 0.59; *p* = 0.766).

**FIGURE 1:**
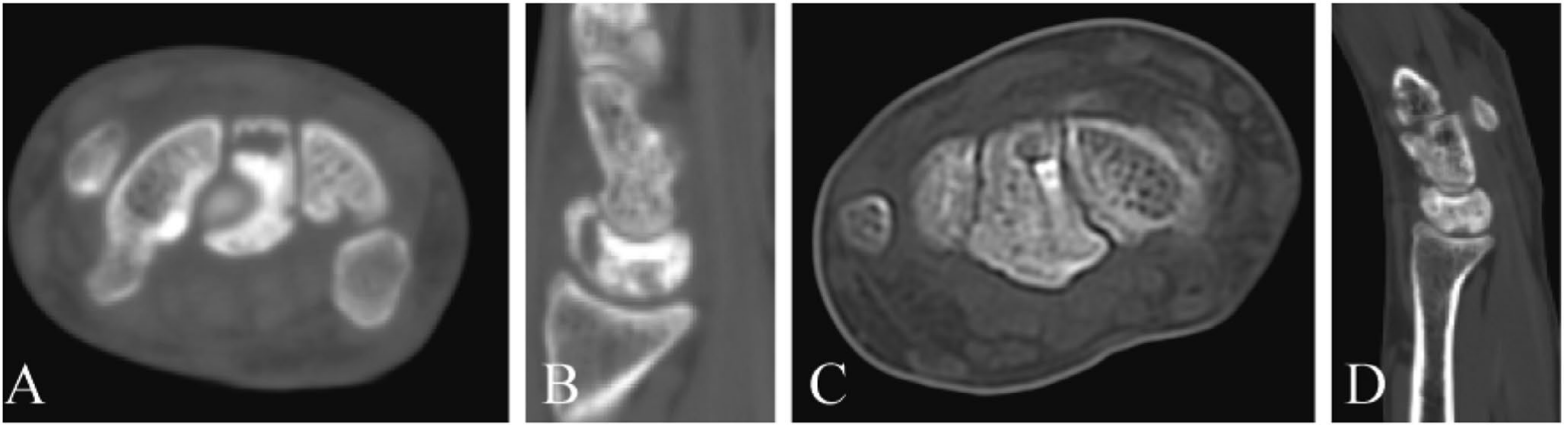
A 29‐year‐old woman presented with right wrist pain for 2 years. Preoperative axial (A) and sagittal (B) CT images demonstrated fracture of the lunate. Postoperative axial (C) and sagittal (D) CT images obtained at 91‐month follow‐up.

**FIGURE 2:**
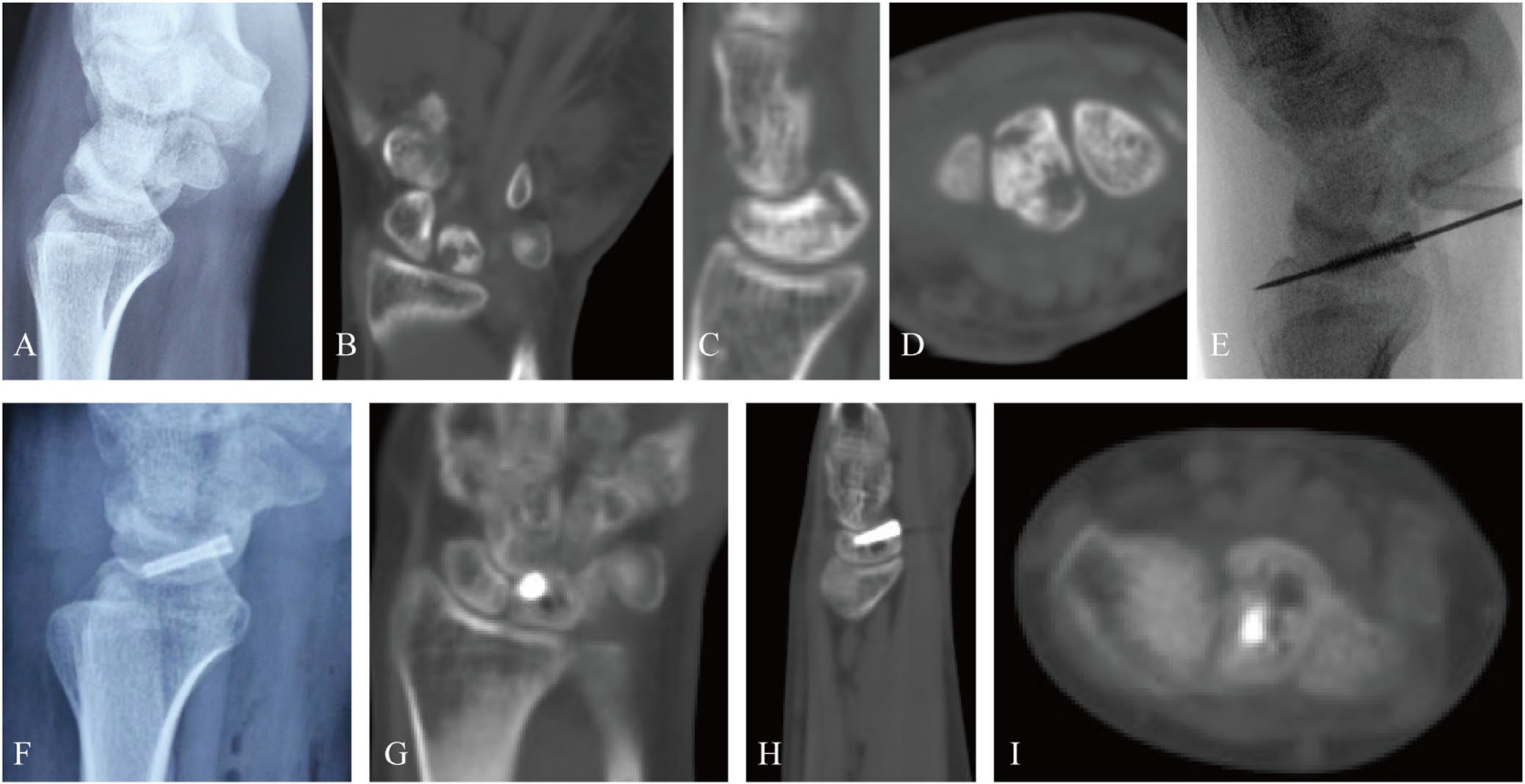
A 31‐year‐old woman complained of left wrist pain for 7 months. Preoperative lateral radiograph (A) and coronal (B), sagittal (C), and axial (D) CT images showed lunate coronal fracture. Intraoperative fluoroscopy (E) demonstrated guidewire placement across the fracture. A lateral radiograph obtained on postoperative Day 2 (F) demonstrated the position of the screw. At 24 months after surgery, follow‐up coronal (G), sagittal (H), and axial (I) CT images showed the healing of the fracture.

**FIGURE 3:**
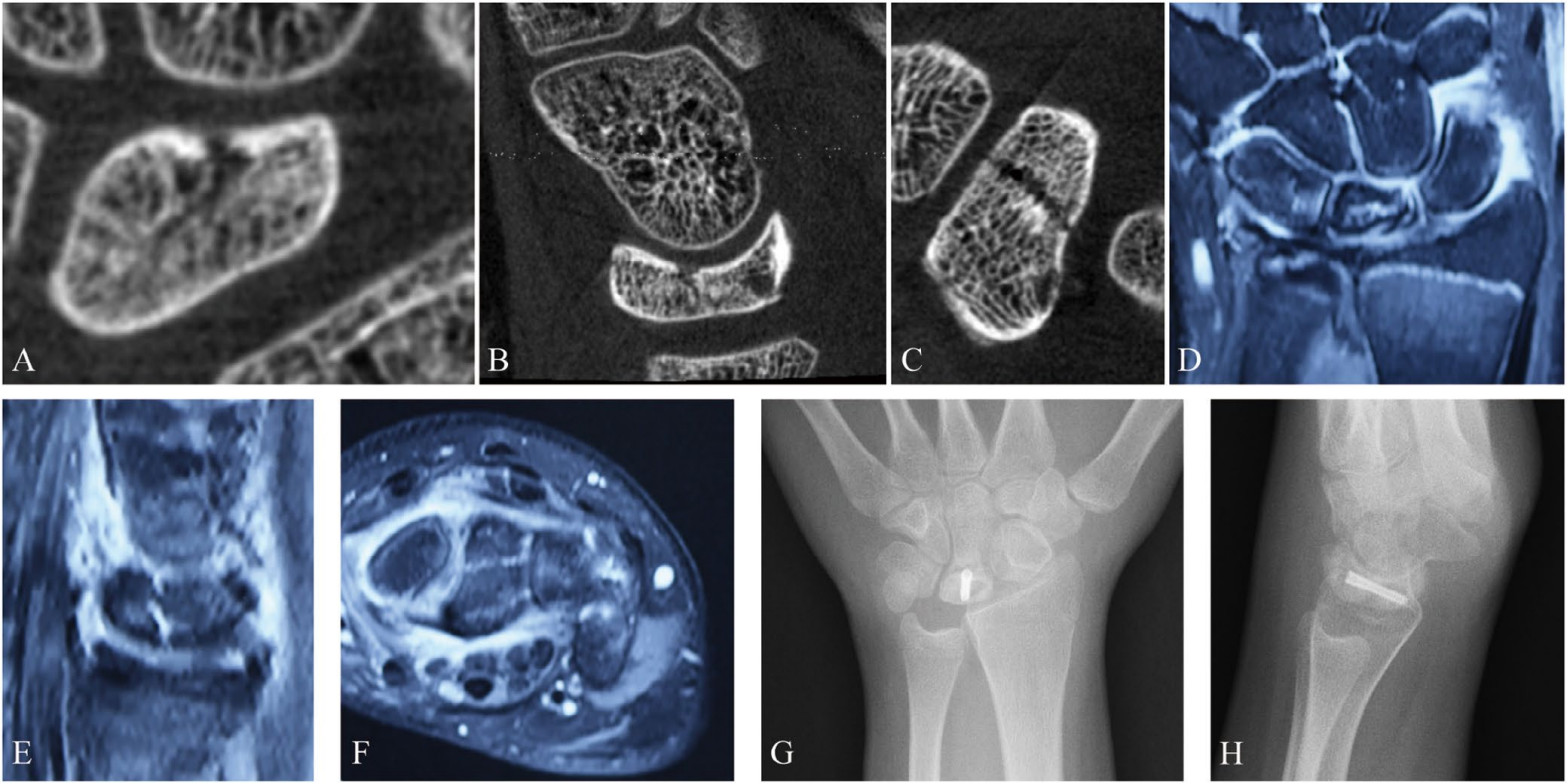
A 16‐year‐old man presented with a 6‐month history of left wrist pain. He initially underwent nonoperative management with immobilization for 4 months, but his symptoms persisted. The lunate coronal fracture was demonstrated on coronal (A), sagittal (B), and axial (C) ultra‐high‐resolution CT images and on coronal (D), sagittal (E), and axial (F) T2‐weighted MRI images. At 27‐month follow‐up, radiographs (G and H) showed satisfactory fracture healing, and the patient was clinically asymptomatic.

## 4 Discussion

The main findings of this study were that headless compression screw fixation alone achieved union of lunate coronal fracture, improved wrist pain and function, and maintained radiographic parameters without progression in Lichtman stage during follow‐up.

### 4.1 Comparison With Existing Treatment Strategies

Currently, the optimal management of Kienböck disease with lunate coronal fractures remains controversial. Lichtman et al. suggested that these fractures do not heal, even after direct or indirect revascularization procedures. Therefore, they advocated excision of the fractured lunate followed by scaphotrapeziotrapezoidal (STT) fusion or proximal row carpectomy [6]. However, in recent years, various reconstructive approaches have been developed in an attempt to achieve bone union. Barrera et al. treated such fractures using a dorsolateral biplane closing radial osteotomy combined with lunate fixation and reported fracture union in eight of 11 wrists [14]. Tagliero et al. reported a union rate of 45% after lunate reconstruction, which was performed with or without vascularized bone grafting [13]. In contrast, Arimitsu et al. found that the union rate after partial capitate shortening varied by fracture location, and none of the coronal fractures achieved union [15]. Rames et al. similarly believed that pedicled vascularized bone grafting procedures were inadequate for treating coronal fractures [17]. These strategies differ in therapeutic principles. Radial or partial capitate shortening osteotomy aims to improve the mechanical environment of the lunate, whereas bone grafting is intended to enhance the local biological conditions. Combined procedures attempt to address multiple aspects simultaneously, but they are more extensive and invasive, and may involve additional surgical morbidity. However, the current literature remains limited and heterogeneous, consisting largely of case reports, with insufficient detail regarding disease severity, fracture morphology, comminution, and arthritic change. As a result, direct comparison across different treatment strategies remains difficult. In our series, all coronal fractures with available imaging at final follow‐up achieved union following headless compression screw fixation, without concomitant bone grafting. This approach may represent a relatively streamlined option while avoiding additional trauma to other donor sites or carpal bones. It should also be noted that the favorable outcomes in our series were not simply attributed to the selection of less severe cases. However, surgeons should be aware that a prolonged period of postoperative immobilization and a long time load‐free exercise may be required, and radiographic consolidation may be delayed. In addition, differences in bone union rates cannot be attributed simply to surgical techniques, because the severity and precise location of the coronal fracture, as well as the presence of sclerosis and cystic changes at the fracture margin, may also affect healing [15].

### 4.2 Possible Biological Mechanism

A key mechanistic question is why coronal fractures of the lunate may still be capable of healing in Kienböck disease without vascularized bone grafting. Although Kienböck disease has traditionally been regarded as avascular necrosis of the lunate, the pathological substrate may not always represent complete diffuse bone death. A previous histopathological study by Xiong et al. demonstrated that, in stage III Kienböck lunates, necrosis was localized mainly around the fracture sites rather than throughout the entire lunate, with a mean necrotic area accounting for only 16.3% of the whole section. Moreover, intralunate vessels remained abundant, and reparative changes, including fibrous granulation tissue, macrophage‐mediated removal of necrotic tissue, osteoid formation, and trabecular remodeling, were still evident. These prior histopathological findings suggest that the lunate may retain some reparative potential even in stage III Kienböck disease, which may represent one possible biological explanation for fracture healing after stable fixation. However, this mechanism was not directly assessed in the present study [18, 19].

### 4.3 Possible Mechanical Mechanism

From a mechanical perspective, the radius of curvature of the proximal lunate articular facet against the distal radius is greater than that of the distal facet articulating with the capitate. This incongruity in curvature between the distal radius and capitate may subject the lunate to a “nutcracker” mechanism, in which concentrated forces across the radiolunate and capitolunate joints act on a structurally weakened bone and predispose it to a coronal split [20]. Once a coronal fracture has occurred, even if it initially involves the trabecular bone, it may progress under continued load if not stabilized promptly [18, 21]. Persistent micromotion between fracture segments may hinder reparative remodeling and vascular ingrowth across the fracture site, thereby contributing to progressive structural failure and fragmentation. In this setting, early and stable internal fixation of the coronal fracture with postoperative load‐free recovery may help to interrupt this process by reducing micromotion and providing a favorable mechanical environment for fracture healing. This represents a plausible mechanical hypothesis for the bone union observed in our series and requires further validation in future studies.

### 4.4 Clinical Implications

Among the 11 patients with available final follow‐up imaging, fracture union was achieved in all cases. In the remaining patient who declined further imaging examinations because of complete symptom resolution, the structural status of the lunate could not be confirmed. Radiographic parameters remained unchanged, and no progression in Lichtman stage was observed in these 11 patients. These findings may suggest radiographic maintenance after fixation; however, this interpretation should be made cautiously. Importantly, the goal of this procedure was not to directly address the vascular problem, but rather to address the structural problem of lunate coronal fracture by achieving fracture union and preventing further structural progression. In this clinical context, fracture union together with the absence of stage progression may be considered a meaningful treatment outcome. Nevertheless, not all coronal fractures in Kienböck disease are appropriate candidates for headless compression screw fixation alone. We agree with Chou et al. that lunates without comminution and without radiocarpal or midcarpal arthritis may be amenable to salvage with internal fixation [12].

### 4.5 Limitations and Strengths

The strengths of this study included its focus on a rare and clinically challenging lunate fracture pattern in Kienböck disease and its evaluation of a relatively streamlined, less invasive surgical approach. In addition, the study included both clinical and radiographic outcomes, with mid‐ to long‐term follow‐up available in most patients.

There are some limitations in our study. First, there is inherent bias in this retrospective study and some clinical data might be incomplete. Second, this is a descriptive case series without a control group, which limits our ability to compare headless compression screw fixation alone with other treatment options for lunate coronal fractures in Kienböck disease. Third, the sample size is relatively small, and the findings should therefore be interpreted with caution. Fourth, accurate screw placement in the lunate requires careful trajectory planning and technical experience. Because all procedures in this series were performed by a senior hand surgeon, the favorable outcomes may partly reflect surgeon expertise. This may limit the reproducibility of this technique among less experienced surgeons. In the future, integration of robotic‐assisted or navigation‐assisted techniques may help improve the precision and reproducibility of screw placement and potentially reduce the learning curve for less experienced surgeons. Fifth, this treatment required a prolonged period of postoperative immobilization; therefore, postoperative rehabilitation protocols may need to be modified to shorten the immobilization time. In addition, although our interpretation is supported by previous histopathological and clinical observations, the biological mechanism underlying fracture union after fixation alone remains inferential. The present study did not directly assess intralunate perfusion or the sequence of revascularization and creeping substitution after fixation.

## 5 Conclusion

In conclusion, the results of our study suggest that headless compression screw fixation alone may be a reasonable treatment option for coronal fractures of the lunate in Kienböck disease in the absence of radiocarpal or midcarpal arthritis and substantial lunate comminution.

## Author Contributions

**Deshui Yu:** investigation, formal analysis. **Binzhi Zhao:** investigation, formal analysis. **Liping Xu:** investigation, formal analysis, supervision. **Ge Xiong:** conceptualization, funding acquisition, supervision, validation, writing – original draft, writing – review and editing. **Jiangchao Zhang:** conceptualization, methodology, writing – original draft, writing – review and editing.

## Funding

This work was supported by the Natural Science Foundation of Beijing Municipality (7222087).

## Disclosure

All authors listed meet the authorship criteria according to the latest guidelines of the International Committee of Medical Journal Editors, and all authors are in agreement with the manuscript.

## Ethics Statement

This study was approved by the institutional review board of Beijing Jishuitan Hospital (K2025186) and written informed consent was obtained from each patient. All procedures performed in studies involving human participants were in accordance with the ethical standards of the institutional and/or national research committee and with the 1964 Helsinki declaration and its later amendments or comparable ethical standards.

## Conflicts of Interest

The authors declare no conflicts of interest.

## Data Availability

The data that support the findings of this study are available from the corresponding author upon reasonable request.
